# Supplementary material for: Smooth Muscle Cell Phenotypic Switch Induced by Traditional Cigarette Smoke Condensate: A Holistic Overview
Source: Int J Mol Sci. 2023 Mar 29;24(7):6431. doi: 10.3390/ijms24076431 (PMC10094728; doi:10.3390/ijms24076431)
Supplement: Supplementary file 1 [file ijms-24-06431-s001.zip › ijms-2249576-supplementary.pdf]

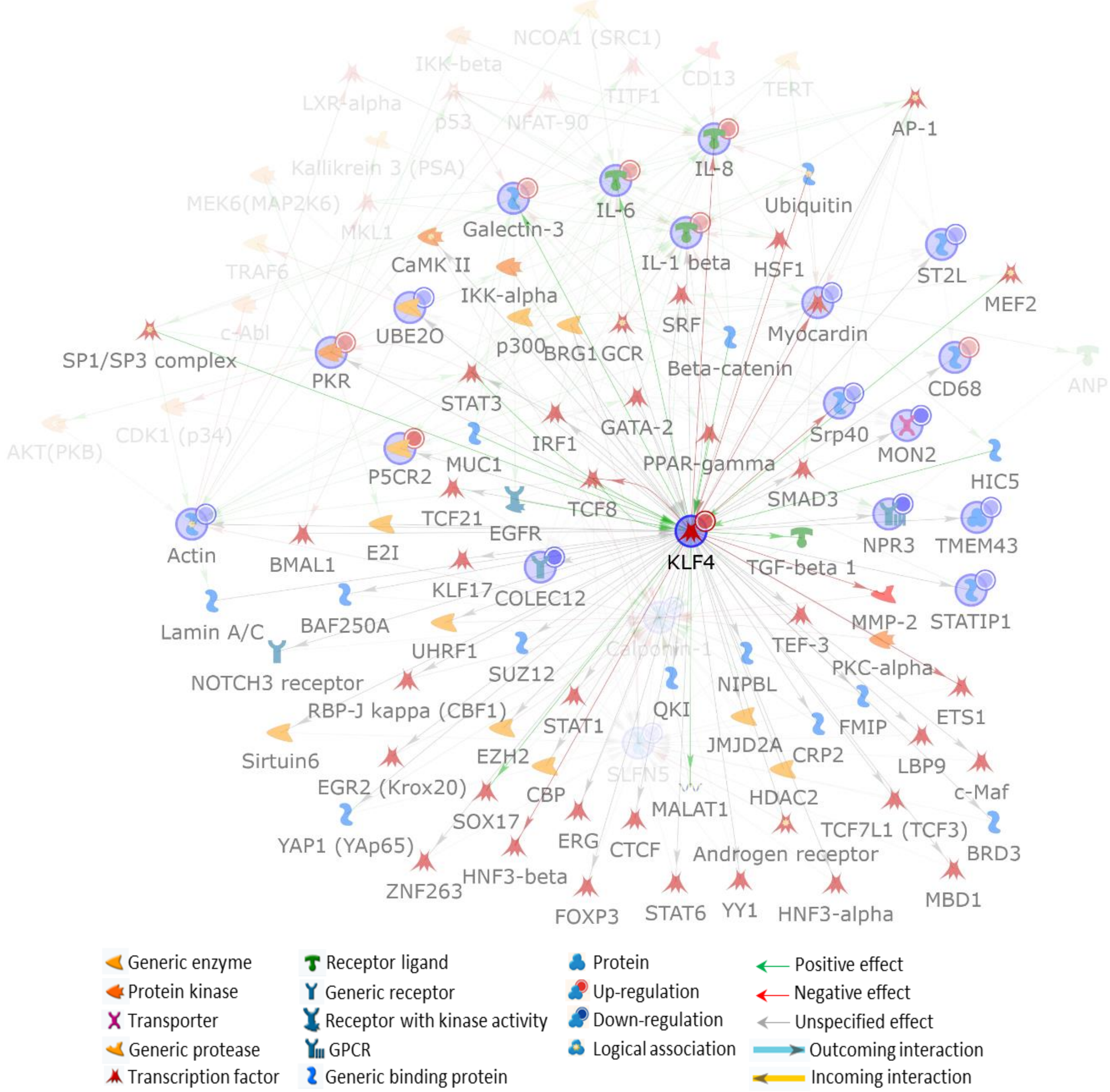

Figure S1. Protein/gene hybrid SPN in KLF4-centred trace-mode.

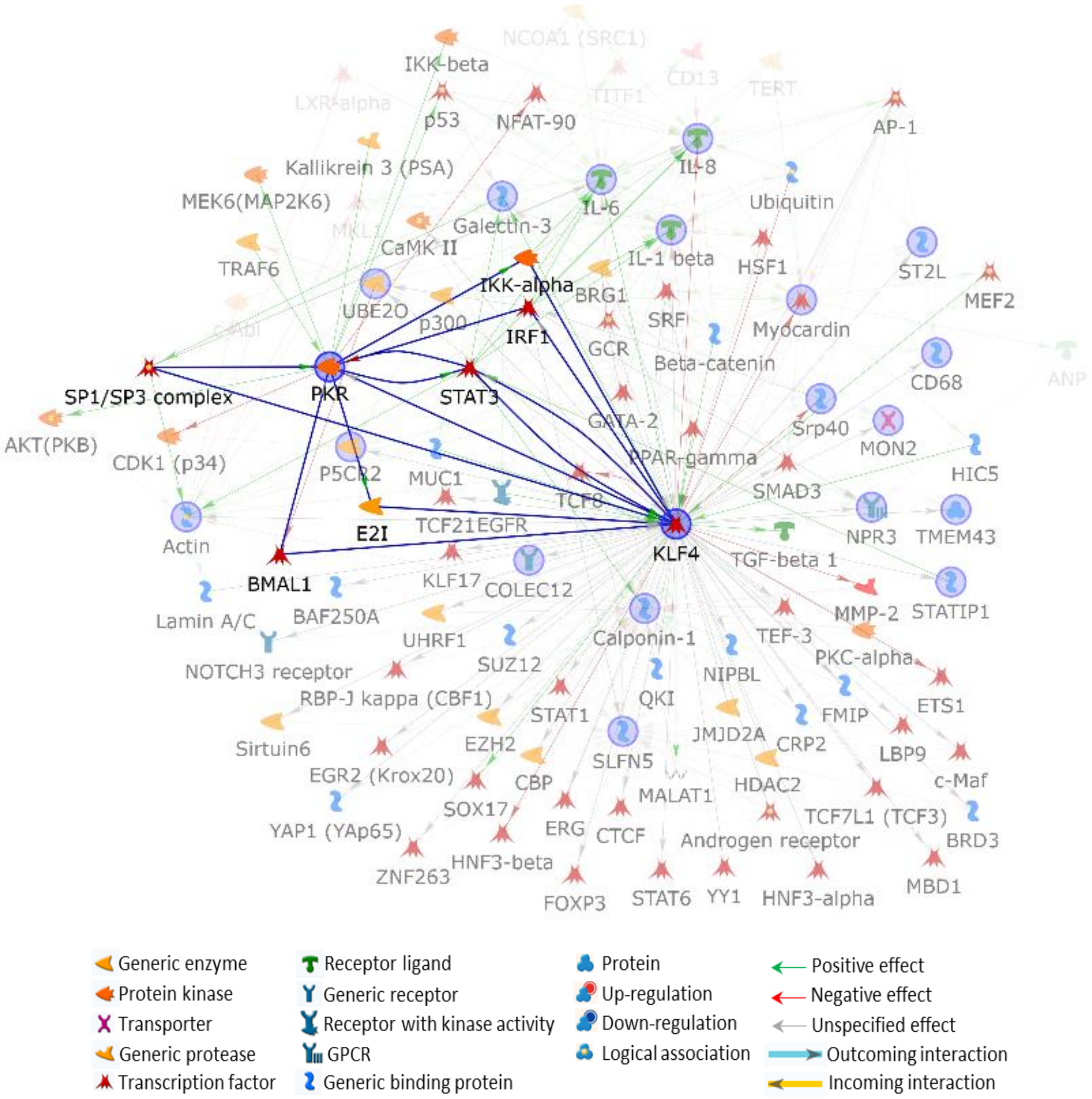

Figure S2. Protein/gene hybrid SPN in KLF4 and PKR centred trace-mode and mark-up view of shortest path (two allowed steps) interconnections (bold in blue) among KLF4 and PKR.
